# The Evolving Trend of Liver Transplantation in Metabolic Diseases: From Origins to Current Perspectives

**DOI:** 10.1002/jimd.70100

**Published:** 2025-10-10

**Authors:** Andrea Pietrobattista, Diego Martinelli, Marco Spada, Carlo Dionisi‐Vici

**Affiliations:** ^1^ Division of Metabolic Diseases and Hepatology Bambino Gesù Children's Hospital IRCCS Rome Italy; ^2^ Division of Hepatobiliopancreatic Surgery, Liver and Kidney Transplantation Bambino Gesù Children's Hospital IRCCS Rome Italy

**Keywords:** improved metabolic control, individualized protocols, inherited metabolic disease, liver transplantation, multidisciplinary management, quality of life

## Abstract

Liver transplantation (LTx) has become, over the years, an increasingly used therapeutic option in patients with inherited metabolic diseases (IMD). Initially performed for Tyrosinemia Type I and ornithine transcarbamylase deficiency, it now accounts as the second indication for pediatric transplants worldwide. The use of LTx has been extended to systemic metabolic disorders, in which a genetically normal liver can correct the defect by providing an enzyme replacement therapy that improves metabolic control and disease burden, reducing the risk of metabolic crises and neurological damage, allowing for the withdrawal, in most diseases, of dietary restrictions and specific medications. The temporal changes, mainly reflecting improved LTx management through a multidisciplinary approach, have provided excellent outcomes and long‐term patient survival, shifting the paradigm from a lifesaving procedure to a life‐improving treatment. However, challenges still exist, particularly, in systemic IMD due to the persistence of the underlying defect in extra‐hepatic tissues. Immunosuppression, especially in organic acidurias, may lead to new, drug‐related, neurotoxic risks. The new indications for transplantation should target endpoints that are not exclusively clinical, addressing major attention to the improvement of health‐related quality of life issues. Protocols for managing LTx in IMD need to be harmonized, and future joint multicenter actions will fill these gaps and provide a uniform vision of this evolving scenario.

**Abbreviations**
ALF: acute liver failure
APLT: auxiliary partial liver transplantation
ASA: argininosuccinic acid
ASL: argininosuccinate lyase
ASS: argininosuccinate synthetase
CNI: calcineurin inhibitor
CNS: central nervous system
CSF: cerebrospinal fluid
DLT: domino liver transplantation
GSD: glycogen storage disease
HCC: hepatocellular carcinoma
ICIMD: International Classification of Inherited Metabolic Disorders
IMD: inherited metabolic disease
IT‐IEM: intoxication‐type metabolic disorders
LDLT: living donor liver transplantation
LKTx: liver‐kidney transplantation
LTx: liver transplantation
MMA: methylmalonic aciduria
MSUD: maple syrup urine
NBS: newborn screening
OA: organic acidurias
OTC: ornithine transcarbamylase deficiency
PA: propionic aciduria
QoL: quality of life
RALF: recurrent acute liver failure
UCD: urea cycle disorders

## 1 Introduction

Liver transplantation (LTx), which represents the gold standard for the treatment of end‐stage liver disease [1], was initially considered as an alternative treatment for inherited metabolic diseases (IMD) in Tyrosinemia Type I and in ornithine transcarbamylase (OTC) deficiency [2, 3]. Starting from those initial approaches, LTx has represented a growing therapeutic option and the evolving indications now encompass a broad range of IMD [4, 5]. Initially, LTx was reserved for those diseases where the metabolic defect, mainly confined to the liver, caused structural hepatic changes or where the potentially fatal outcome, as seen in neonatal urea cycle disorders (UCD), could have been counteracted by the replacement of the missing enzymatic activity [2, 3]. Its positive impact is now evident also for diseases in which toxic metabolites, originating from a defective enzymatic activity in multiple organs and systems, can freely interchange through the systemic circulation. In this setting, a genetically normal liver can correct the defect by providing an enzyme replacement therapy which improves the patient's metabolic control and disease burden, reducing the risk of metabolic crises and of neurological damage [4, 6]. Over the years, LTx became an established therapeutic approach in IMD becoming the second indication for pediatric LTx worldwide, accounting for nearly 20% of pediatric LTx in Europe, 15% in Australia and New Zealand and up to 30% in the United States of America [7, 8]. The temporal changes were more relevant in pediatrics rather than in the adult setting, mainly reflecting recent updates on pre‐ and posttransplant management.

In this sense, the evolution of surgical technique has been crucial, and, particularly, the affirmation of partial liver transplant methods, initially reduced liver and subsequently split liver transplantation and living donor transplantation [9]. This has significantly increased the number of organs available for transplant and consequently the possibility of expanding the indications, without penalizing patients with end‐stage liver diseases.

The American Scientific Registry for Transplant Recipients documented this evolving trend analyzing 30 years (1987–2017) of transplant activity in IMD [4]. Data from the registry showed a steady increase over the years of children treated by LTx for UCD and glycogen storage disease (GSD) Type 1, while for other diseases, such as organic acidurias (OA) and maple syrup urine disease (MSUD), LTx became an established indication increasing from 0% to nearly 10% throughout the 3 decades. Recent European multicenter studies confirmed these trends, providing an overview of frequency and current practice on liver and/or liver and kidney transplantation in amino and organic acid‐related disorders [6, 10].

LTx results in IMD are usually excellent, with a 1‐year survival of 97% in a large registry in the United States, while long‐term survival exceeding 80% has been described in other experiences [5, 6, 7, 8, 11, 12, 13].

Regardless of the underlying metabolic defect, the transition from being an IMD patient to a liver transplant recipient carries potential transplant‐related complications (e.g., vascular/biliary problems, rejection, and PTLD), whose burden is, however, lower than the natural history of the disease without transplantation. Ultimately, to predict the potential impact of LTx it is crucial to have a comprehensive understanding of disease pathophysiology and of metabolic compartmentalization [14].

**FIGURE 1:**
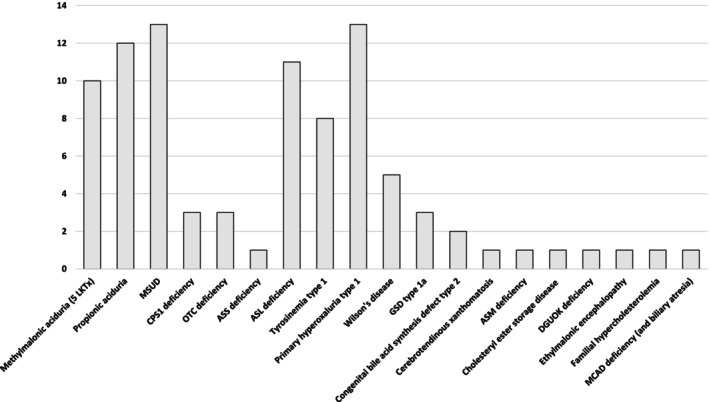
Case series of liver transplantation performed at Bambino Gesù Children Hospital in metabolic patients in the years 2010–2025, with an overall survival rate at follow‐up exceeding 96%.

This manuscript provides an overview of the evolving role of LTx in IMD and also reflects the authors' center's experience, based on about a hundred LTx in IMD (Figure 1), which represents 26% of the total pediatric liver transplant activity and in whom we observed a 10‐year patient survival exceeding 90%.

## 2 Liver Transplantation in IMD

### 2.1 Classification of IMD Potentially Leading to Liver Transplantation

**FIGURE 2:**
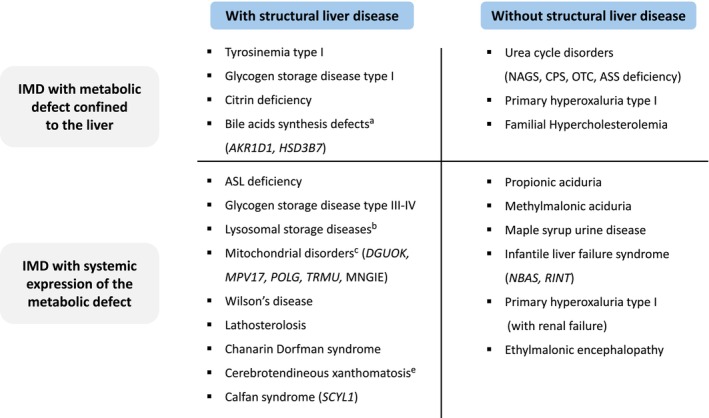
Inherited metabolic diseases potentially eligible for liver transplantation. Classification is based on structural liver abnormalities and systemic expression of the metabolic defect. ^a^Not responsive to bile acid replacement therapy; ^b^acid lipase and sphingomyelinase deficiency presenting with liver failure or unresponsive to enzyme replacement therapy; ^d^selected cases on the bases of genotype; ^e^severe progressive cholestatic form leading to liver failure.

The classification of metabolic diseases has historically been a complex task, given the vast spectrum of associated conditions. The recently introduced International Classification of Inherited Metabolic Disorders (ICIMD), which includes over 1400 disorders, categorizes metabolic conditions based on their biochemical and functional characteristics [15]. From a LTx perspective, it appears preferable to cross‐reference ICIMD with a clinically driven categorization [4, 7]. On these bases, IMD suitable for LTx may be divided into four categories, considering the presence or absence of liver structural abnormalities and then further classified based on the hepatic and extrahepatic expression of the metabolic defect, with or without the involvement of other organs and tissues [4]. Using this methodological approach, the list of IMD eligible for LTx has undergone timing adaptations, given the growing knowledge on the impact of LTx and the increasing availability of novel therapies. Figure 2 shows an updated list of IMD potentially treatable by LTx.

### 2.2 Indications to Liver Transplant in IMD

The indications for LTx in IMD are more complex than those considered in the conventional setting of nonmetabolic conditions and are based upon a different and wider intention‐to‐treat model (Table 1).

**TABLE 1:** The table illustrates the indications for liver transplantation in patients with metabolic diseases and the recommended timing.

Indication	Disease	When
Prevent/reduce risk of metabolic decompensation	MSUD Organic acidurias Urea cycle defects	Early
Improve metabolic control	MSUD Organic acidurias Urea cycle defects GSD 1	Early
Prevent/reduce risk of organ complications	MMA (CKD, eye disease, and pancreatitis) PA (CMP, eye disease, and pancreatitis) FH (cardiovascular disease)	
Improve long‐term neurological outcome	MSUD Organic acidurias Urea cycle defects	Early
Improve growth	MSUD Organic acidurias Urea cycle defects GSDs	Early
Improve disease burden	All diseases	Early
Improve quality of life	All diseases	Early
Prevent/treat ALF/RALF	BASDs NBAS deficiency SCYL1 deficiency (?) Tyrosinemia Type 1* Urea cycle defects Wilson's disease	
Treat end‐stage liver disease	ASL deficiency BASDs Citrin deficiency Lathosterolosis LSDs Chanarin Dorfman syndrome Cerebro tendineous xanthomatosis Wilson's disease	
Treat liver cancer	Tyrosinemia type 1 Citrin deficiency GSDs MMA	Late
Attempt to treat an untreatable disease (selected cases)	Ethylmalonic encephalopathy DGUOK deficiency MPV17 deficiency POLG deficiency TRMU deficiency MNGIE	Early

Abbreviations: ALF: acute liver failure; BASD: bile acid synthesis disorders; FH: familial hypercholesterolemia; GSD: glycogen storage disease; LSD: lysosomal storage disease; MMA: methylmalonic aciduria; MNGIE: mitochondrial neurogastrointestinal encephalopathy; MSUD: maple syrup urine disease; PA: propionic acidemia; RALF: recurrent acute liver failure.

LTx is increasingly considered an elective treatment in life‐threatening illnesses such as severe early onset forms of MSUD and UCD, providing a metabolic cure through the replacement of the enzymatic defect [16]. At the same time, it plays the role of a lifesaving procedure for IMD with functional and structural liver defects presenting as acute liver failure (ALF) or chronic liver injury with a nonnegligible risk of hepatocellular carcinoma (HCC). The presence of a liver mass—either with confirmed HCC or strong clinical suspicion—represents a major indication for LTx, particularly, in late‐treated Tyrosinemia Type I and GSD Type I. Remarkably, the significant impact of newborn screening (NBS) is supported by the continuous decreased rate of LTx need for HCC in Tyrosinemia Type 1 [17]. Neonatal diagnosis allowed early nitisone start, providing effective prevention of life‐limiting liver disease and long‐term safety [18]. LTx is now reserved only for the few patients with ALF unresponsive to nitisinone, for those with HCC or, less frequently, to treat an end‐stage liver disease [19]. Accordingly, at our center, eight LTxs were performed for Tyrosinemia Type I at a median age of 10.9 years, seven due to HCC, one for liver failure and none of these patients were diagnosed through NBS. In addition, the use of LTx for ALF and end‐stage liver disease spans a wide and increasing range of IMD, all involving hepatic parenchymal damage but differing in the metabolic patho‐mechanisms. Well‐documented examples include Wilson's disease and UCD, alongside rarer conditions like lathosterolosis, cholesteryl ester storage disease, acid lipase deficiency, and citrin deficiency or the rapidly progressive cholestatic form of cerebrotendinous xanthomatosis [20, 21, 22, 23, 24].

In some lysosomal storage diseases, such as Niemann–Pick disease type C and acid sphingomyelinase deficiency, where a severe/acute early‐onset liver disease may anticipate the appearance of neurological signs, the indication for LTx remains controversial since liver involvement does not reliably predict the neurological severity, which remains the primary clinical burden [25, 26, 27]. The limited window time for diagnostic evaluation, combined with the frequent lack of genotype/phenotype correlation, makes the option of LTx a real decision challenge, provided that allocating donor organs for an incurable neurodegenerative condition may not be the most effective use of resources.

In ALF of indeterminate etiology, rapid decision‐making is essential. In such settings, customized next‐generation sequencing panels, or whole‐exome sequencing could provide timely diagnosis and guide transplant decisions [28, 29]. To this regard, within the broad spectrum of conditions causing ALF, recurrent acute liver failure (RALF) has been increasingly associated with inherited disorders affecting the machinery of cellular trafficking [30]. These disorders result from mutations in genes regulating intracellular transport and endoplasmic reticulum homeostasis, impairing Golgi‐to‐ER retrograde transport and disrupting hepatocyte stress responses. As a result, the liver becomes highly vulnerable to metabolic decompensation, particularly during febrile episodes, which serve as a primary trigger [30]. Among the over 350 genes which have been linked to intracellular trafficking defects in human disease [31], three have been directly associated with RALF: infantile liver failure syndrome Types 2 and 3, caused by pathogenic variants in *NBAS* and *RINT1*, respectively, and CALFAN syndrome linked to *SCYL1* mutation [30]. These three conditions lack specific biomarkers and often progress rapidly, making LTx a potentially suitable therapeutic option or a preventive intervention. However, posttransplant outcomes vary, with some patients achieving hepatic stability and others showing neurological deterioration or immune dysregulation, thus raising concerns regarding the long‐term neurological and immunological outcomes [30, 32, 33, 34, 35].

Beyond disorders with structural liver involvement or presenting as ALF, the improvement of metabolic instability has become one of the major indications to transplant metabolic patients when medical and dietary management fail to prevent recurrent metabolic decompensations leading to neurological damage, multiorgan failure, or death. In this setting, LTx represents an increasingly used elective option in intoxication‐type metabolic disorders (IT‐IEM), such as UCD, MSUD, and OA, to provide better metabolic control, reduce disease burden, and improve quality of life (QoL) [4, 6, 36, 37, 38, 39, 40, 41]. Although there is still no consensus on post‐LTx management, in the majority of centers, UCD patients discontinue dietary protein restrictions, nitrogen scavengers, and arginine/citrulline supplements, being free from hyperammonemia crises [16]. At long‐term follow‐up, glutamine levels remain in the normal range while, depending on individual diseases, patients with proximal defects [e.g., *N*‐acetylglutamate synthetase, carbamoyl phosphate synthetase I, and OTC deficiency] maintain reduced plasma citrulline levels whereas those with distal cytosolic defects (e.g., argininosuccinate lyase [ASL] and argininosuccinate synthetase [ASS]deficiency) still present elevation of citrulline and argininosuccinic acid (ASA), although at lower levels compared to pre‐LTx [41, 42].

**FIGURE 3:**
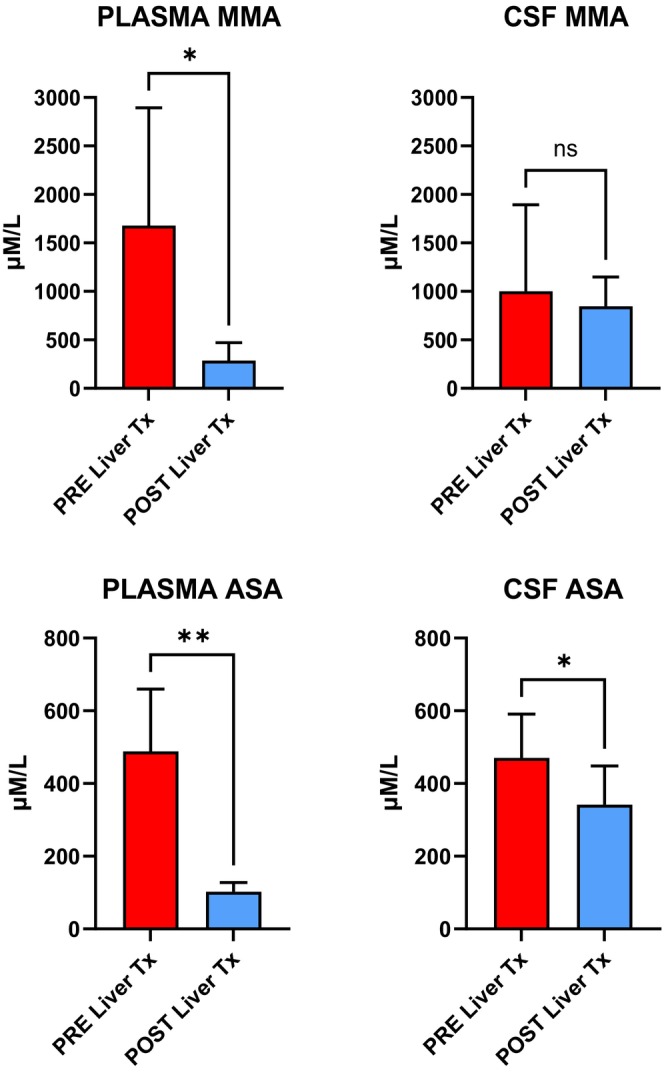
Disease specific biomarker changes in patients treated by liver transplantation. Top panel shows methylmalonic acid (MMA) concentration in plasma and CSF in 6 patients with methylmalonic aciduria before and after LTx. Bottom panel shows argininosuccinic acid (ASA) concentration in plasma and CSF in nine patients with ASL deficiency before and after LTx. Graphs are obtained using data reported in references [37, 41].

The option of transplantation has long been debated in ASL deficiency due to the fear of neurological complications and of unfavorable cognitive outcome [40, 43]. However, recent findings from our center showed stabilization or improvement in neurological function post‐LTx [41]. Early transplantation (i.e., < 5 years) was associated with significant improvement of developmental/cognitive scores and maturation of executive functions, which corresponded to the improvement of brain atrophy at MRI. Biochemically, ammonia and glutamine levels normalized in all patients, and plasma ASA significantly declined. In cerebrospinal fluid (CSF), although showing reducing trend values, ASA levels remained elevated. (Figure 3).

In MSUD, LTx has emerged as an elective transformative treatment, offering significant metabolic and neurological benefits [36, 44, 45]. Post‐LTx, patients can follow a normal dietary regimen without protein restriction. The clinical course is unremarkable with stabilization/improvement of the neurological disease, the levels of branched‐chain amino acids remain in a safe range, and alloisoleucine became only borderline detectable [45, 46]. Symptomatic hyperleucinosis during febrile illness has been anecdotally reported in both related and unrelated donor transplantation and, in this light, a seek day regimen during febrile illness, especially if associated with diarrhea and dehydration, is still recommended [47, 48].

In OA, although preexisting neuro‐disability does not get reversed, LTx may improve neurological function, especially when performed at a younger age, but its long‐term impact still needs to be evaluated through tailored longitudinal evaluations [6, 38, 49]. Metabolic crisis and neurological decline can still occur due to the persistence of the enzymatic defect in the central nervous system (CNS), which is not replaced by LTx. Studies have reported post‐LTx metabolic strokes, a lack of neurological improvement, and progression of neurodegeneration [37, 50, 51, 52]. Similar to what is seen in ASL deficiency, early transplanted methylmalonic aciduria (MMA) patients also showed significantly improved developmental/cognitive scores and executive functions, along with improvement of cortical thickness and white matter maturation indexes at brain MRI [37]. One out of the six MMA patients reported in the study had a mild post‐LTx stroke‐like episode, but the characteristic MRI changes disappeared at follow‐up. Metabolic monitoring comparing pre‐ and post‐LTx sampling showed that methylmalonic acid levels significantly improved in plasma, while they remained substantially unchanged in CSF. (Figure 3) Differently, biomarkers of mitochondrial dysfunction (i.e., lactate, alanine, and related ratios) significantly improved in CSF post‐LTx [37].

In OA, the prevention and treatment of late organ complications, in particular chronic kidney disease in MMA and cardiac dysfunction in PA, are the other recognized indications for transplantation in later ages [38]. In MMA, when renal involvement progresses to end stage kidney disease, combined liver and kidney transplant (LKTx) is associated with better outcomes than isolated kidney transplant [53]. Combined LKTx is a complex procedure which provides better renal function preservation compared to isolated kidney transplant through a greater reduction of posttransplant methylmalonic acid concentrations, whose long‐term accumulation may lead to new kidney injury [54].

Cardiomyopathy and prolonged QTc interval are two life‐threatening complications observed in about 40% of PA patients [55]. Some reports demonstrated reversal of cardiomyopathy after LTx, suggesting that cardiomyopathy might be an elective indication for LTx [56, 57, 58]. Nevertheless, reoccurrence of cardiomyopathy after LTx has been reported [59].

Overall, LTx is now a valuable option for IT‐IEM. Early LTx should be first considered in young children with poor metabolic control, a high disease burden to reduce the risk of severe neurological sequelae [38]. This approach gained evidence through different studies that demonstrated the benefit of LTx (beyond the mere patient/graft survival) in reducing the risk of acute decompensations, improving neurodevelopment, allowing dietary liberalization, lowering healthcare costs, and enhancing QoL [5, 6, 8, 36, 37, 38, 39, 41, 60, 61]. Notably, the evaluation of QoL in IT‐IEM should be assessed using specifically designed tools, such as MetabQoL 1.0, which better capture disease burden and the impact of LTx than general instruments like PedsQL [39]. This extended approach is essential to strengthen the benefit of LTx by linking neuropsychological monitoring with psychosocial support, helping patients and families to develop effective coping strategies for stress and anxiety.

Similarly, LTx has become a treatment option also in selected GSD Type 1a cases, especially those with onset of severe hypoglycemia in the neonatal period or in the first weeks/months of life, requiring an intense high carbohydrate dietary regimen provided through continuous tube or gastrostomy feeding to prevent hypoglycemia [62]. After LTx, GSD Type 1a patients reach a physiological fasting tolerance with a normal dietary regimen [63]. Moreover, other GSD—including GSD Type 1b, III, IV, VI, and IX—are eligible for LTx, to improve metabolic control, for the presence of liver neoplasms (adenomas or HCC), or as treatment for end‐stage liver disease [63]. In GSD Type 1b, the incidence of rejection necessitating titration of immunosuppression was relatively high (73%), but the recent introduction of SGLT2 inhibitors was effective in reducing post‐LT infectious and gastrointestinal morbidities, with no adverse effects noted [64]. In GSD Type IIIa, an adult patient developed after‐LTx worsening clinical signs of myopathy, along with increasing levels of transaminases and creatine kinase, while in GSD IV, although LTx has increased the life expectancy and QoL, it did not alter the progression of the extrahepatic manifestations, especially cardiomyopathy [65, 66].

As for our own experience, all patients with UCD and MSUD discontinued protein restriction, OA patients increased protein intake within the RDA recommendations, all withdrew single amino acid supplementation, ammonia scavengers were interrupted in UCD, nitisinone in tyrosinemia patients, while carnitine therapy was maintained in OA. Similarly, hypoglycemia disappeared in GSD 1a patients with no further need for nutritional supports, which corresponded to a relevant improvement in QoL.

Despite improvement in the outcomes and reduced risk related to surgery, there are conditions where LTx remains controversial due to the lack of knowledge on natural disease progression and on genotype/phenotype correlations. A paradigmatic example is provided by mitochondrial hepatopathies, which encompass a wide range of systemic disorders caused by mutations in nuclear or mitochondrial DNA that can manifest as early‐onset primary liver dysfunction [67, 68]. Traditionally, mitochondrial disorders have been considered a contraindication to LTx, presenting complex challenges, particularly, concerning the frequent development of severe neurological manifestations with survival rates that overcome the expected posttransplant benefits [69]. Studies have reported variable outcomes, with survival rates often influenced by the specific genetic mutations and the presence of extrahepatic manifestations. A literature review reported single case or small series with positive outcomes in very selected patients; however, the overall survival rate post‐LT for mitochondrial hepatopathies is approximately 30%, primarily due to progressive neurological deterioration [67]. Nevertheless, there are few examples showing that in selected patients, LTx may represent a suitable therapeutic option as seen in MINGIE and in DGUOK deficiency [70, 71]. Moreover, LTx has been successfully attempted in ethylmalonic encephalopathy, a mitochondrial‐related disease with invariable fatal outcomes, allowing long survival and the change from a severe encephalopathy to a milder disease phenotype [72].

Overall, in the metabolic and transplant community, there is a clear paradigm shift on the role of LTx from a lifesaving to a life‐improving treatment. These advances have occurred in parallel with the availability of novel therapeutical options along with the widespread use of NBS. The latter might offer the opportunity to perform an early transplantation in diseases with a stormy course, like MSUD, UCD, and OA, or to prompt the initiation of life‐saving therapies, as seen in Tyrosinemia Type I, which strongly reduces the risk of late complications and the need for LTx. On the other side, the advent of novel drugs greatly influenced LTx indications in some IMD, as in primary hyperoxaluria. Combined liver and kidney transplantation in primary hyperoxaluria type I has represented the primary treatment option for limiting organ morbidity from systemic oxalosis [73]. The recent development of an RNA interference therapy, lumasiran, showed in a Phase III clinical trial to be effective in decreasing urinary oxalate in affected children [74], thus reducing the need for organ transplantation for those patients with no or late access to the new treatment. Other examples of IMD eligible for LTx with emerging treatment options include familial hypercholesterolemia, Wolman disease, acid sphingomyelinase deficiency, and arginase deficiency [75, 76, 77, 78].

### 2.3 Metabolic Management of Liver Transplantation in IMD: Toward a Model of Precision Medicine

The management of patients with IMD undergoing LTx presents distinct peculiarities compared to the most common pool of LTx recipients and requires in a great proportion of cases a multidisciplinary approach, not limited to transplant surgeons, metabolic and hepatology clinicians, but extended to a range of professional disciplines (e.g., anesthetists, cardiologists, intensive care team, nephrologists, neurologists, neuroradiologists, psychologists and dieticians), who are essential for continuous monitoring of transplant progress forming the basis for a precision medicine approach. This is, particularly, evident in the growing group of patients with IMD with systemic involvement who cannot be completely cured by LTx. In such cases, it is essential the definition of individualized protocols adapted to each disease (and to each patient), with the aim of establishing a model of precision medicine, with particular emphasis on the intraoperative and immediate postoperative phases. At these times, patients are potentially exposed to metabolic decompensation triggered by stressors, such as fasting, anesthesia, surgery, ischemia reperfusion, fluid and electrolyte imbalances, or infections. This may promote in OA, UCD, and MSUD a catabolic state with increased endogenous protein turnover and the risk of life‐threatening crises due to the accumulation of toxic metabolites not yet clearable from the newly implanted liver. Detailed indications for perioperative intravenous fluids (e.g., glucose, lipids), pharmacotherapy prescriptions (e.g., carnitine, ammonia scavengers), nutritional regimen and monitoring of metabolic testing during and after surgery are mandatory to avoid metabolic complications [6, 37, 38, 41, 54]. In the immediate post‐LTx period, a key element to consider is the recovery of graft function. If this recovery is rapid and adequate, it will allow the discontinuation of pretransplant metabolic therapies and nutritional restrictions. Conversely, in cases of delayed graft function, these steps may be postponed, and dialysis treatment may become necessary in case of persistent metabolic abnormalities. Ultimately, precise integration of transplant‐related monitoring with disease‐specific metabolic testing is essential. This ensures that care is dynamically adapted to the patient's evolving clinical condition and supports optimal long‐term outcomes within a personalized and multidisciplinary framework. At long‐term follow‐up, targeted on individual disease characteristics, longitudinal clinical, neurological, biochemical, and imaging monitoring provide the support for understanding the impact of LTx in modifying disease course and patient's outcome.

### 2.4 Immunosuppressive Therapy and Posttransplant Neurotoxicity

While LTx ameliorates the primary metabolic derangements in IMD, the introduction of immunosuppressive agents, particularly, calcineurin inhibitors (CNIs), like tacrolimus and, to a lesser extent, cyclosporine, introduces new drug‐related potential neurotoxic risks, reported in 20% of adult recipients and 8% of pediatric patients [79]. Clinically, CNI‐induced neurotoxicity manifests from tremors, confusion, and headaches to severe complications such as seizures, cerebral hemorrhage, ischemic stroke, central pontine myelinolysis, and posterior reversible encephalopathy syndrome [79, 80, 81, 82]. The pathophysiology is multifactorial, with mitochondrial dysfunction, disruption of the blood–brain barrier, and neuronal excitotoxicity potentially involved. In IMD patients, preexisting neurological impairment, mitochondrial vulnerabilities, and altered pharmacokinetics may amplify the neurotoxic effect of immunosuppression. OA, and particularly MMA [83], represents a disease category at higher risk of CNI‐induced neurotoxicity, raising the need for a timely differential diagnosis when sudden neurological symptoms, mimicking classical metabolic stroke‐like events, appear after transplantation. Since the two etiologies are clinically indistinguishable, an MRI scan together with specific metabolic determination in plasma and CSF should be performed to promptly reduce/discontinue tacrolimus therapy in case of CNI‐induced neurotoxicity [37]. mTOR inhibitors, such as sirolimus and everolimus, provide an alternative to CNIs in mitigating posttransplant neurotoxicity [54, 84, 85, 86].

### 2.5 Surgical Aspects in Liver Transplantation in IMD

Surgical advancements have significantly improved LTx outcomes in IMD, reaching almost zero perioperative mortality and expanding eligibility [87]. Early referral for transplantation is emphasized, and living donor liver transplantation (LDLT) is increasingly utilized, particularly, in regions with limited deceased donor availability [88]. Although concerns exist regarding heterozygous donors, long‐term studies have demonstrated successful metabolic outcomes [4, 12, 89, 90]. Among the few circumstances that require special caution and careful donor evaluation, OTC deficiency, which is inherited in an X‐linked manner, should be considered as an exclusion criterion for LDLT for heterozygous female donors [91]. To this regard, hyperammonemia has been reported following successful LDLT using a heterozygous maternal donor graft because of lower OTC activity [92].

In those countries where, like Italy, there is an efficient system of deceased donor transplantation and mandatory split liver policies have been adopted, all pediatric patients with metabolic diseases can be transplanted, with a percentage of dropouts from the waiting list that is almost zero [10, 93]. In this setting, LDLT contributes to a percentage of transplants that varies from 10% to 25% (https://unos.org/; https://www.anzlitr.org/; https://www.eltr.org).

The allocation of deceased donor livers is based on the pediatric end‐stage liver disease score (PELD), which estimates the risk of death on the waiting list of patients with end‐stage liver disease. To provide transplant opportunities to children with metabolic diseases without liver damage, the definition of appropriate scores and list status was essential [10]. From a strictly surgical point of view, transplantation in patients with metabolic diseases, especially those without liver damage, has specific peculiarities that must be considered. These patients do not have portal hypertension and tolerate portal and systemic clamping less during hepatectomy, and therefore this must be as short as possible to ensure hemodynamic and metabolic stability. Unlike patients with chronic liver disease who have organomegaly and ascites, in the case of metabolic diseases, the donor‐recipient dimensional matching, understood as graft‐to‐recipient weight ratio, must be more restrictive to avoid conditions of large‐for‐size graft that increase the risk of vascular complications and primary nonfunction or early allograft dysfunction. This is, particularly, true in cases of neonatal ALF in which the available space in the abdominal cavity is limited, and it is necessary to use hyperreduction or monosegment transplant techniques [94].

In the case of transplantation from a deceased donor, the donor characteristics and the ischemia times must be such as to ensure a prompt functional recovery of the transplanted organ to reduce the risk of acute metabolic decompensations in the immediate posttransplant period. Perfusion machines can significantly contribute, even for split liver procedures, to reducing ischemia–reperfusion injury and therefore to increasing the number of usable organs and improving outcomes [95, 96].

The first series of transplants in children with metabolic diseases was burdened by a higher incidence of vascular complications, especially hepatic artery thrombosis [7]. Arterial reconstruction in these patients can be more difficult, especially in the case of left lateral segment transplantation with only the left hepatic artery, since the recipients do not have compensatory hypertrophy of the hepatic artery. The use of microsurgical anastomosis techniques and, in the case of split liver, the division of the arterial axis while maintaining the celiac tripod with the left lateral segment has contributed to reducing the incidence of arterial thrombosis [97, 98].

Thanks to the domino transplantation technique, LTx in patients with metabolic diseases without liver damage can theoretically be a transplant with a zero balance in terms of deceased donor organs used or with a positive balance if the metabolic patient is transplanted from a living donor.

The term domino liver transplantation (DLT) refers to a specific technique in which the explanted structurally and functionally normal liver from a metabolic patient (except for the underlying enzymatic defect) is used for a recipient unaffected by the donor's disease [99]. Main indications for DLT were primary hepatic malignancy, viral, and alcoholic cirrhosis. MSUD is the best‐known indication for DLT, with proven long‐term safety and efficacy [100, 101] Notably, a recent study described the possibility of using MSUD livers for DLT also in patients with other metabolic diseases [PA (2 pts), ASS deficiency, GSD Type 1 and GSD Type 3] reporting disease cross‐correction and positive clinical results, with only one death in the ASS patient due to surgical complications of the transplant [102]. Less commonly, metabolic livers used as domino grafts were from patients with acute intermittent porphyria, hereditary fibrinogen A‐chain amyloidosis, hyperhomocysteinemia, PA, MMA, familial hypercholesterolemia, primary hyperoxaluria, and hemophilia A [103, 104, 105, 106]. However, not all the non‐MSUD grafts used for DLD were associated with good outcomes, as some recipients developed early or late symptoms and even organ failure related to the underlying defects. Therefore, beside MSUD, when a metabolic graft may lead to the development of acquired enzymatic deficiency, DLD should be limited to: (a) patients with unresectable primary or metastatic liver malignancies, with a life expectancy estimated to be shorter than the interval until the development of metabolic symptoms; (b) urgent LTx, as a bridge to permit a secondary LTx. It is important that potential candidates for domino transplantation are fully informed of the potential risks of disease transmission coming from the domino grafts.

Auxiliary partial liver transplantation (APLT), which consists of implanting orthotopically a healthy partial liver graft after partial hepatectomy of the native liver [107], has been reported in PA and ASS Deficiency Type I, but its use is still controversial [108, 109]. A potential benefit of APLT is represented by the possibility of preserving the native liver while waiting for future liver‐targeted gene therapy interventions, besides providing safety in case of graft dysfunction. Technical key issues are the graft venous outflow, the site and type of vascular anastomoses, and the regulation of portal flow to avoid the steal phenomenon that may cause graft dysfunction or failure [110].

Domino and auxiliary transplantation can be used together. In the first case, a domino graft from a child with Wilson's disease and a second graft from a child with OTC deficiency were successively implanted into a woman with familial amyloidosis [111]. This approach theoretically eliminates the risk of a single domino developing symptoms related to the underlying enzymatic defect. In fact, the two domino grafts with different metabolic liver diseases could compensate for each other's metabolic defects. After that, the concept of domino cross‐auxiliary LTx was developed, using noncirrhotic metabolic grafts for auxiliary transplantation in other patients with an alternative type of metabolic disease. Also, two partial livers were exchanged between two patients with different noncirrhotic metabolic diseases. In this way, LTx was performed without a healthy living donor or a deceased donor [111, 112, 113].

### 2.6 Unanswered Questions and Unmet Needs

Despite significant advancements in LTx for metabolic diseases, several unanswered questions and unmet clinical needs persist, especially for those diseases in which the enzymatic defect is expressed at a systemic level. To this regard, a critical gap lies in the knowledge of CNS metabolic compartmentalization, as shown in MMA and in ASL deficiency by monitoring posttransplant the concentration of disease biomarkers in CSF, which revealed no significant reduction compared to what is seen at plasma levels [37, 41]. These recent findings shed light on the unmet need of exploring new therapies targeting the CNS as adjunct treatments to LTx, or to novel gene‐ and mRNA‐related therapies, which have the liver as their exclusive target tissue [114, 115, 116, 117]. However, since LT precludes the enrollment in these novel experimental therapeutical approaches, this issue should be explicitly addressed at the time of LT enrollment. Unanswered questions concern the limited knowledge of the underlying mechanisms causing cardiomyopathy in PA or optic neuropathy and sensorineural deafness in PA and MMA, highlighting the further issue of the role and timing of LTx in their prevention and treatment. Immunosuppressive therapy represents another challenge given the higher risk of CNI‐induced neurotoxicity, potentially exacerbating preexisting neurological impairment in some metabolic conditions.

## 3 Conclusion

One of the main objectives of this review was to show the evolution and temporal changes for the use of LTx in metabolic diseases, moving from the initial indication aimed at the replacement of a structurally damaged organ to the treatment of diseases with systemic expression of the metabolic defect, with the aim of modifying the clinical course and improving patients' prognosis. The new indications for transplantation should take into account endpoints that are not exclusively clinical, addressing major attention to the impact of LTx in improving the QoL.

Protocols for managing LTx in IMD still need to be harmonized. Future joint multicenter actions, combined with translational research addressed to LTx, such as ex vivo normothermic perfusion of metabolic livers [118], will represent the most effective way to fill these gaps and to provide a uniform vision of this evolving scenario.

## Author Contributions

All authors contributed to the writing and/or revision of the manuscript, and all authors have read and approved the submission of this manuscript.

## Conflicts of Interest

The authors declare no conflicts of interest.

## Data Availability

The data that support the findings of this study are available on request from the corresponding author. The data are not publicly available due to privacy or ethical restrictions.
